# Supplementary figures and images for: From Early Morphometrics to Machine Learning—What Future for Cardiovascular Imaging of the Pulmonary Circulation?
Source: Diagnostics (Basel). 2020 Nov 25;10(12):1004. doi: 10.3390/diagnostics10121004 (PMC7760106; doi:10.3390/diagnostics10121004)

## Slide 1
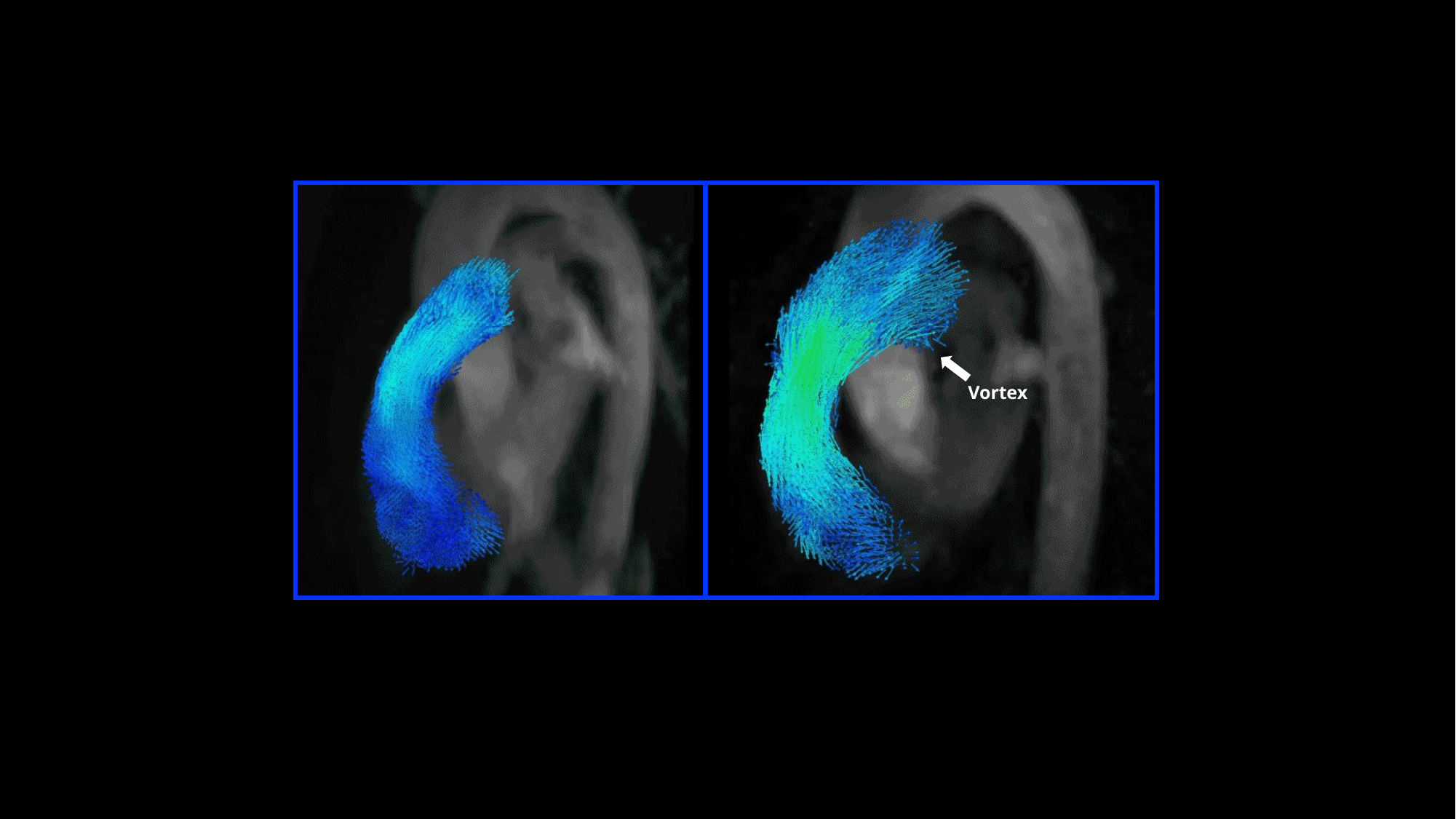

Vortex

Supplement: Supplementary file 1 [file diagnostics-10-01004-s001.pptx]
